# Food Intake and Satiety Response after Medium-Chain Triglycerides Ingested as Solid or Liquid

**DOI:** 10.3390/nu11071638

**Published:** 2019-07-17

**Authors:** Tyler Maher, Alistair Sampson, Magdalena Goslawska, Cristina Pangua-Irigaray, Amir Shafat, Miriam E Clegg

**Affiliations:** 1 Oxford Brookes Centre for Nutrition and Health, Faculty of Health and Life Sciences, Oxford Brookes University, Oxford OX3 0B, UK; 2 Physiology, School of Medicine, National University of Ireland, Galway H91 TK33, Ireland; 3 Department of Food and Nutritional Sciences, University of Reading, Whiteknights, Reading RG6 6AP, UK

**Keywords:** MCT, dietary fat, energy intake, appetite, gastric emptying, food form

## Abstract

Consuming medium-chain triglycerides (MCT) may reduce subsequent energy intake and increase satiety compared to long-chain triglycerides (LCT) but this may be dependent on the physical form in which MCT is ingested. Twenty-nine participants completed four trials where they consumed a breakfast containing either LCT or MCT in solid (Con-S and MCT-S, respectively) or liquid (Con-L and MCT-L, respectively) form. Appetite ratings and gastric emptying (GE) were taken at baseline and at 15 min intervals for 4 h. Energy intake was assessed at an *ad libitum* meal and via weighed food records for the remainder of the day. *Ad libitum* energy intake was highest in Con-L (4101 ± 1278 kJ vs. Con-S, 3323 ± 1196; MCT-S, 3516 ± 1058; MCT-L, 3257 ± 1345; *p* = 0.001). Intake over the whole day was significantly lower in MCT-L (7904 ± 3244) compared to Con-L (9531 ± 3557; *p* = 0.001). There were significant differences in GE times (*p* < 0.05), with MCT breakfasts delaying GE to a greater extent than LCT, and MCT-L having the longest GE times. There were no differences in appetite sensations. MCT reduce subsequent intake without affecting subjective sensations of appetite when consumed in liquid form.

## 1. Introduction

Medium-chain triglycerides (MCT) are triglycerides with fatty acid chain lengths of 6–12 carbon atoms long, which includes capronic acid (C6:0), caprylic acid (C8:0), capric acid (C10:0) and lauric acid (C12:0) [1]. Unlike traditional long-chain triglycerides (LCT), MCT are absorbed quickly through the portal venous system and transported directly to the liver where they undergo *β*-oxidation [2]. This accelerated transport is due to MCT not requiring bile salts for emulsification, or carnitine palmitoyl transferase to cross the mitochondrial membrane, and thus they are absorbed more like glucose than fat [3]. The rapid absorption has also been linked to increased satiety, as MCT are fully absorbed at the point of ingestion, as opposed to LCT, where some remain in the intestinal lumen until subsequent food intake [4]; and also due to the production of the ketone *β*-hydroxybutyrate which has been shown to be anorexigenic [5] due to the sparing of glucose [6].

There is evidence that MCT ingestion results in greater satiety compared to LCT [7,8,9,10,11], however this is not a universal finding [12,13]. There are clear differences in study design which may influence the effect MCT has on satiety, such as the dose of MCT, and the time between MCT ingestion and the *ad libitum* meal. 

A potential study design difference which may interfere with the satiating properties of MCT is the vehicle used for the MCT bolus. The majority of studies that report an effect of MCT administered it in a liquid meal [10,11], preload [7,14], or as part of a liquid infusion [15]; whereas the studies that incorporated MCT as part of a solid food found no difference between LCT and MCT [12,13]. The research group of St-Onge and colleagues [13] conducted two studies investigating the effect of MCT on satiety; one utilizing a solid breakfast consisting of muffins which contained 20 g of MCT, and one where a yoghurt containing an extra 10 g of MCT was consumed 3 h after the original muffin breakfast. They found that energy intake after the MCT preload was significantly reduced, whereas the muffins containing MCT had no effect on energy intake. Whether this is due to the increased dosage, the time between preload and *ad libitum* meal, or the fact that MCT was consumed in a liquid food is not known. This study was designed to examine the difference in satiety response between MCT meals given in liquid form and solid form and compare the difference to LCT meals given in liquid and solid form. 

## 2. Materials and Methods 

### 2.1. Design

All participants completed four trials in a random order where they consumed a breakfast consisting of chocolate brownie, ice cream and milk, or the same breakfast blended into a smoothie. The lipids were baked into the brownies which acted as vehicle for the lipids. Data were collected over 4 h, during which time the participants rested in the laboratory. At the end of the data collection period, participants consumed an *ad libitum* buffet meal, after which they left the laboratory and recorded all food intake in a weighed food diary for the remainder of the day. Trials were on non-consecutive days, separated by a minimum of 48 h and a maximum of 10 days. 

### 2.2. Participants

Inclusion criteria were as follows: 18–60 years of age, weight stable for the three months leading up to commencement of the study, taking no medication which could affect appetite, unrestrained eaters (as determined by the Three-Factor Eating Questionnaire [16] and Dutch Eating Behavior Questionnaire [17]), and with a body mass index (BMI) of 18.5–29.9 kg·m^−2^. After institutional ethical approval (UREC: 181183, approved by the Oxford Brookes University Research Ethics Committee on 23rd March 2018), participants (M: 13, F: 16, age: 26 ± 6 years, weight: 69.7 ± 17.2 kg, height: 172 ± 10 cm, BMI: 24.0 ± 2.9 kg·m^−2^, body fat: 23.6 ± 8.1%) who were recruited through social media, posters and through a research activity mailing list, completed a medical questionnaire and gave their written informed consent (Supplementary Material S1). Based on recommendations from Blundell and colleagues stating 25 participants is adequate to detect a 10% difference in energy intake, 30 participants were recruited to allow for drop out, and 29 finished the study.

### 2.3. Standardization

In the 24 h preceding the first trial, participants were required to record all food and drink consumed. This diary was replicated in the 24 h preceding the remaining trials. Physical activity and alcohol were to be avoided in this 24 h period and participants were asked to keep caffeine intake at habitual levels. Participants were asked to travel to the laboratory using the same method on each test day and upon arrival they were asked if there were any differences in the standardization period.

### 2.4. Protocol

Participants attended a preliminary screening session at the Oxford Brookes Centre for Nutrition and Health (OxBCNH), where all experimental trials took place. Height was measured (to the nearest 0.1 cm) using a mobile stadiometer (Seca 217, Seca, Birmingham, UK), and body mass and body fat percentage (to the 0.1 kg and 0.1%, respectively) were measured using a body composition monitor (BC-418 MA, Tanita, Amsterdam, Netherlands). If eligible based on BMI and BF% criteria, participants completed the Three-Factor Eating Questionnaire [16], Dutch Eating Behavior Questionnaire [17], and a medical history questionnaire. Participants were provided with commercially available digital weighing scales (Color Match Digital Scale, Argos, Milton Keynes, UK) and instructions on how to complete the standardization booklet. 

Each trial consisted of one visit to the laboratory, where participants arrived between 7:00–9:00 am after a 10–12 h overnight fast. All trials for each participant started at the same time. Participants rested for 10 min in a seated position before giving baseline samples, after which they were provided with the breakfast. Participants were instructed to eat this within 10 min, after which the data collection period began. Immediately upon completion of the breakfast, participants rated the palatability of the breakfast on visual analogue scales (VAS). Participants rested in the laboratory for 4 h whilst measures of gastric emptying (GE)and subjective sensations of appetite were taken. At 4 h, participants consumed an *ad libitum* buffet lunch until satiation, after which they were free to leave the laboratory. Participants were instructed to complete weighed food diaries for the remainder for the day. 

### 2.5. Test Breakfast 

The test breakfast consisted of a chocolate brownie, ice cream and milk. This was consumed either as a solid food (Con-S and MCT-S) or blended into a liquid milkshake (Con-L and MCT-L). All brownie ingredients were purchased in a local supermarket and made to identical portions (Tesco Stores ltd, Cheshunt, UK; 736.7 kJ (174.6 kcal) 3.2 g fat, 30.4 g carbohydrate, 4.9 g protein), to which ~853 kJ of oil was added. In order to achieve an isocaloric amount of added lipid, the control oil was 23.06 g vegetable oil (Con; rapeseed oil, Tesco Stores Ltd., Cheshunt, UK), and the test oil was 25.00 g MCT oil (MCT; Muscleform, Norfolk, UK, (caproic acid 2%, caprylic acid 50–60%, capric acid 30–45% and lauric acid 3%)), both of which were added to the brownie recipe before cooking. The brownie was served with 40 g vanilla ice cream (Walls, London, UK; 331.2 kJ (78.8 kcal) 3.0 g fat, 12.0 g carbohydrate, 1.0 g protein) and 300 g semi-skimmed milk (Tesco Stores ltd, Cheshunt, UK; 817.3 kJ (194.4 kcal) 7.0 g fat, 18.7 g carbohydrate, 14.1 g protein). The entire breakfast contained 2738 kJ (655.3 kcal) 36.3 g fat, 61.1 g carbohydrate and 20.0 g protein. Pilot testing indicated this portioning was palatable and a suitable portion size. In Con-L and MCT-L the brownie, ice cream and milk were mixed using a food blender (NutriBullet, Bullet Brands, Australia) for 3 × 10 s mixes, with five s between each mix to allow the mixture to settle. This was done immediately before serving to avoid separation of the oils. 

### 2.6. Gastric Emptying

GE was measured by adding 100 mg ^13^C octanoic acid (Euriso-top, Paris, France) to the breakfast which resulted in ^13^CO_2_ in the breath after rapid and complete oxidation in the liver. Breath samples were collected in duplicate by blowing gently into a 12 mL exetainer (Labco, Lampete, UK) through a drinking straw and immediately replacing the lid upon withdrawal of the straw, every 15 min for 3 h. The rate of appearance of ^13^CO_2_ was measured using isotope ratio mass spectrometry (ABCA, Sercon Ltd., Crewe, UK) and expressed relative to Vienna Pee Dee Belemnite (V-PBD), an international standard of known ^13^C composition. Carbon dioxide production rates were estimated to be 5 mmol CO_2_·min^−1^·m^−2^ body surface area [18] and body surface area calculated from height and weight according to Haycock et al. The ^13^CO_2_ enrichment curve and CO_2_ production were used to calculate emptying from the stomach according to Ghoos et al. [19] and Schommartz et al. [20]. Results are displayed as the half time (Thalf), lag phase (Tlag), latency time (Tlat), and ascension time (Tasc) in minutes. “Half time” is the time required to empty 50% of the ingested meal, “latency” is the initial delay of the cumulative exhalation curve, “lag” is time taken to reach maximal ^13^CO_2_ exhalation rates which is equivalent to the time of the inflection point of the curve, and “ascension” refers to the high rates of ^13^CO_2_ exhalation between the lag-phase and half time. 

### 2.7. Subjective Sensations

Hunger, fullness, desire to eat (DTE), prospective food consumption (PFC) and nausea were assessed using 100 mm visual analogue scales (VAS) where 0 mm indicated “not at all” and 100 mm indicated “extremely”. These were recorded at baseline and every 15 min until the *ad libitum* buffet lunch.

### 2.8. Food Intake

Four h after consumption of the breakfast, participants were presented with an *ad libitum* buffet lunch consisting of three plates of sandwiches. Prior to testing, participants chose three different sandwiches from a list, which were used for all four trials. Sandwiches were matched for energy content, and the lunches consisted of two portions of each sandwich cut into quarters. Participants were given 20 min to eat in isolated booths in OxBCNH and were told to eat until “comfortably full and satisfied”. Sandwiches were given in excess of expected consumption, and each of the three plates of sandwiches was covertly weighed before serving and at the end of the *ad libitum* lunch to ascertain food and energy intake. Energy intake in the free-living environment was assessed with weighed food records for the remainder of the study day. 

### 2.9. Statistical Analysis

Data was analyzed using SPSS v.25 software for windows (SPSS inc. Somers, NY, USA). Area under the curve (AUC) values for appetite sensations were calculated using the trapezoidal rule. Data were checked for normality using the Shapiro-Wilks test, and were log transformed if appropriate before undergoing statistical analysis. Mauchly’s test for sphericity was used to check the assumption of sphericity. Outliers were assessed using studentized residuals. Differences in energy intake (kJ) at the *ad libitum* lunch, in the free-living environment, and total intake over the whole day (sum of the breakfast, *ad libitum* lunch, and free-living energy intake), as well as all GE parameters, were analyzed using two-way repeated measures ANOVAs (oil x food form). Where a significant interaction was identified, a *post hoc* one-way ANOVA and Bonferonni-adjusted pairwise comparison were made. An ANCOVA with the baseline as a covariate was used to examine differences in subjective sensations across the four trials. Pearson’s correlation coefficient was used to examine the relationship between energy intake and GE, energy intake and nausea, and GE and nausea. Data are expressed as means and SD. Significance was accepted at the alpha level of *p* < 0.05. 

## 3. Results

### 3.1. Energy Intake

**Figure 1:**
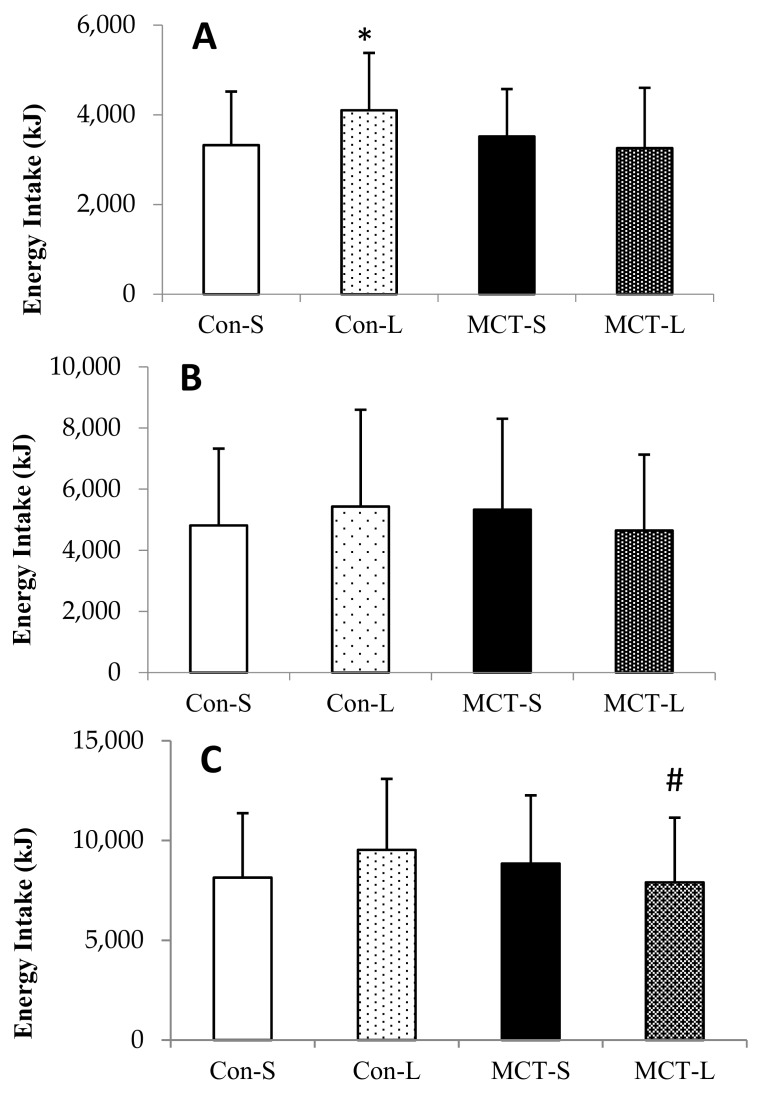
Mean and individual energy intake (kJ) at the *ad libitum* meal (**A**), free-living environment (**B**) and total over the whole day (**C**). Data are expressed as means and vertical bars indicate standard deviation. * Denotes a significant difference compared to all other trials, # denotes a significant difference compared to control oil in liquid form (Con-L). Significance was accepted at the *p* < 0.05 level.

There were small significant main effects for oil (*F*_(1,28)_ = 8.715, *p* = 0.006, *d* = 0.26) and for food form (*F*_(1,28)_ = 4.687, *p* = 0.039, *d* = 0.21), as intake was reduced by an average of 325.9 ± 1247.3 kJ in MCT compared to LCT trials and a reduction of 259.5 ± 1245.9 kJ in solid trials. There was a significant interaction of oil and food form on energy intake at lunch (*F*_(1,28)_ = 16.340, *p* < 0.001, *η*^2^ = 0.369). *Post hoc* testing identified that energy intake in Con-L was higher by 779.6 ± 1237.0 kJ than in Con-S (*p* = 0.001, *d* = 0.63), 585.9 ± 1168.9 kJ than in MCT-S (*p* = 0.008, *d* = 0.17), and 844.8 ± 1311.4 kJ than in MCT-L (*p* = 0.003, *d* = 0.64). There were no significant differences between any of the other trials (all *p* > 0.05; Figure 1A).

There were outliers in Con-S, Con-L and MCT-L for food record data, as identified by studentized residuals of 3.42, 3.99 and 3.06, respectively. The analysis was repeated with outliers removed, and this did not affect the outcome of the statistical tests, and so original tests were run with outliers included. There was no interaction of oil and food form on energy intake during the rest of the day (*F*_(1,28)_ = 1.671, *p* > 0.05, *η*^2^ = 0.056), nor were there any main effects of oil (*F*_(1,28)_ = 0.319, *p* > 0.05, *η*^2^ = 0.011) or food form (*F*_(1,28)_ = 0.16, *p* > 0.05, *η*^2^ = 0.001; Figure 1B). 

There was a non-significant trend for reduced intake after MCT breakfasts compared to LCT breakfasts (*F*_(1,28)_ = 3.016, *p* = 0.094, *η*^2^ = 0.104), and no main effect of food form on total intake (*F*_(1,28)_ = 0.007, *p* > 0.05, *η*^2^ = 0.006). However, there was a significant interaction of oil and food form on intake, as MCT-L resulted in lower energy intake than MCT-S (7904 ± 3244 kJ vs. 8846 ± 3416 kJ) whereas LCT-L led to greater intake than LCT-S (9532 ± 3557 kJ vs. 8137 ± 3237 kJ; *F*_(1,28)_ = 10.099, *p* = 0.004, *η*^2^ = 0.280). *Post hoc* testing showed energy intake was significantly lower in MCT-L compared to Con-L by 1627.7 ± 3400.5 kJ *(p* = 0.001, *d* = 0.48); there were no other significant differences between any trials, although there was a trend for reduced intake in Con-S compared to Con-L (*p* = 0.073; Figure 1C).

### 3.2. Gastric Emptying

Analysis of the results indicated data for three participants were outliers (one in Con-L and two in MCT-S). Transforming these into natural log did not alter their status as outliers. Due to the fact they were unlikely to be genuine results as the data did not fit normal GE curves; these were removed from the final analysis. The data were 367%, 159% and 114% greater than the mean values before their removal from the data. Data are presented in Table 1. 

There were significant effects of oil on Thalf (*F*_(1,25)_ = 56.281, *p* < 0.001, *d* = 0.49), Tlag (*F*_(1,25)_ = 44.206, *p* < 0.001, *d* = 0.86), Tlat (*F*_(1,25)_ = 11.336, *p* = 0.002, *η*^2^ = 0.312; *p* = 0.008 before removal of outliers) and Tasc (*F*_(1,25)_ = 42.177, *p* < 0.001, *η*^2^ = 0.628). MCT breakfasts delayed Thalf by 37.4 ± 38.5 min, Tlag by 10.5 ± 12.2 min, Tlat by 5.6 ± 15.9 min, and Tasc by 36.4 ± 41.5 min compared to LCT breakfasts. There was a small significant main effect of food form (*F*_(1,25)_ = 6.524, *p* = 0.017, *d* = 0.36) on Tlag, as liquid breakfasts accelerated Tlag by 4.7 ± 13.1 min compared to solid breakfasts. Similarly, there was medium main effect (*F*_(1,25)_ = 8.866, *p* = 0.006, *η*^2^ = 0.262) on Tlat, with faster Tlat after liquid breakfasts compared to solid breakfasts (40.6 ± 11.2 vs. 50.4 ± 18.8 min). Thalf was not significantly different in the solid (145.5 ± 35.0 min) compared to the liquid (147.7 ± 49.4 min; *F*_(1,25)_ = 0.38, *p* > 0.05, *d* = 0.24), or Tasc (*F*_(1,25)_ = 2.096, *p* > 0.05, *η*^2^ = 0.077), with only 11.9 ± 44.7 min difference between the means.

There was a statistically significant interaction between oil and food form (*F*_(1, 25)_ = 9.367, *p* = 0.005, *η*^2^ = 0.273; *p* = 0.188 before removal of outliers). *Post hoc* tests showed that Thalf was significantly delayed in MCT-S compared to Con-S (*p* = 0.034, *d* = 0.71) and Con-L (*p* < 0.001, *d* = 1.06), and it was significantly delayed in MCT-L compared to Con-S (*p* = 0.002, *d* = 0.93) and Con-L (*p* < 0.001, *d* = 1.21), but whereas there was a trend for delayed Thalf in Con-S compared to Con-L (*p* = 0.067, *d* = 0.35), there was no difference between MCT-S and MCT-L (*p* > 0.05, *d* = 0.37).

There was also a significant interaction of oil and food form on Tlag, as MCT-S and MCT-L times were virtually identical, whereas Tlag in Con-L was quicker than in Con-S (*F*_(1,25)_ = 7.253, *p* = 0.012, *η*^2^ = 0.225). Tlag was faster in Con-L compared to all other trials (Con-S: *p* = 0.007, *d* = 0.67; MCT-S: *p* < 0.001, *d* = 1.43; MCT-L: *p* < 0.001, *d* = 1.21), and there was a trend for faster Tlag in Con-S in comparison to MCT-S (*p* = 0.083, *d* = 0.57).

There was a significant interaction effect of oil and food form on Tasc (*F*_(1,25)_ = 8.194, *p* = 0.008, *η*^2^ = 0.247). MCT-L delayed Tasc compared to Con-S and Con-L (*p* = 0.001, *d* = 0.56 and *p* < 0.001, *d* = 1.06, respectively), and there was a non-significant trend of delayed Tasc in MCT-L compared to MCT-S (*p* = 0.051, *d* = 0.59). MCT-S, however, was only significantly different to Con-L (*p* < 0.001, *d* = 0.67).

### 3.3. Subjective Sensations

**Figure 2:**
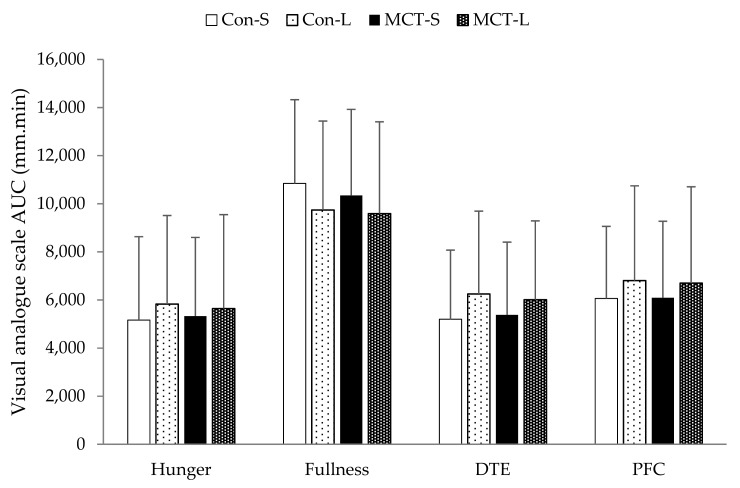
Area under the curve values for hunger, fullness, desire to eat (DTE) and prospective food consumption (PFC) following breakfasts in all trials. Data are expressed as means and vertical bars indicate standard deviation.

No differences in AUC for hunger (*F*_(4,111)_ = 0.102, *p* > 0.05, *η*^2^ = 0.003), fullness (*F*_(4,111)_ = 0.461, *p* > 0.05, *η*^2^ = 0.012), DTE (*F*_(4,111)_ = 0.498, *p* = 0.685, *η*^2^ = 0.013), PFC (*F*_(4,111)_ = 0.408, *p* > 0.747, *η*^2^ = 0.011), or nausea (*F*_(4,111)_ = 0.915, *p* = 0.436, *η*^2^ = 0.024; Figure 2) were observed.

## 4. Discussion

To our knowledge, this is the first study to examine whether the form in which MCT are delivered influences satiety. This study has found that MCT results in greater satiety and reduces subsequent food intake at an *ad libitum* lunch and over the whole 24 h period. A liquid meal containing LCT increased energy intake by 15% compared to its solid LCT counterpart, and MCT provided in liquid form resulted in a non-significant decrease in overall energy intake by 11% over the course of one day compared to the same meal consumed in solid form. The liquid MCT meal also led to a 17% reduced energy intake compared to the isocaloric LCT meal. This may be mediated by a delaying of GE, as MCT breakfasts led to delayed half, lag, latency and ascension times compared to LCT breakfasts. 

The results of the current study corroborate previous investigations that examined the effect of MCT on subsequent energy intake. The group of Rolls [7] were the first to examine the satiating properties of MCT incorporated into liquid preloads consumed 30 minutes before a meal, and found intake was reduced by 5%, 8% and 19% after 100 kcal, 200 kcal, and 300 kcal of MCT compared to an isocaloric load of LCT. Similarly, results from our own laboratory show a 14% reduction in energy intake after a smoothie containing 858 kJ (205 kcal) of MCT compared to LCT [11]. In the current study, there was no significant difference at the group level in energy intake at the *ad libitum* lunch between solid or liquid meals containing MCT, despite inspection of individual data showing 20 out of 29 participants consuming less after the MCT-L meal. Energy intake was significantly higher at the *ad libitum* lunch after a liquid LCT meal compared to a solid LCT meal, with 24 out of 29 participants consuming more after the liquid LCT meal. There was no difference between solid LCT and solid MCT meals, but intake after the liquid MCT meal was significantly lower than after the liquid LCT meal. Given that solids are generally more satiating than liquids [21], these results demonstrate the satiating properties of MCT when administered in liquid food/beverages. This decrease may have clinical significance for weight management, as 100 kcal has been cited as the deficit needed to produce meaningful results at the population level [22]. Thus, our results indicate a liquid breakfast containing MCT shows promise as a method of reducing overall energy intake over the course of the entire day. 

Bloom, Chaikoff and Reinhardt [23] found that, due to their smaller molecular weight, MCT are not incorporated into chylomicrons like LCT are, instead diffusing through the intestine and travelling to the liver via the portal vein where they are oxidized. The absorption of MCT has been reported to be as fast as glucose [24]. In spite of this, we report that MCT delays GE compared to LCT. This corroborates previous findings from our laboratory where MCT delayed GE compared to butter and olive oil [25], and early work showing that long-chain fatty acids were more effective at slowing gastric emptying than fatty acids of 10 or fewer carbon atoms [26]. Furthermore, despite previous evidence showing that liquid meals are emptied quicker than solid meals [27], there was no difference in GE parameters between solid and liquid MCT meals, whereas Con-L led to a significantly quicker lag phase and latency phase compared to Con-S as would be expected. This delay in GE may explain the decreased subsequent intake with MCT consumed in liquid form compared to LCT in liquid form but no difference between intake in the solid trials. Marciani et al. [28] found that blending a meal containing both solid and liquid components delays gastric emptying by removing the gastric sieving component of the meal; where the liquid part empties quickly and leaves the solid to be broken down. This means the bulk of the meal is retained in the stomach and is emptied at a slower rate. Previous work from our laboratory has confirmed these findings using soup as a meal [29], and so this removal of the gastric sieve is a feasible explanation for the delayed GE in liquid trials, however this only occurred in the MCT-L trial and it does not explain the similarity between solid and liquid MCT trials. Duodenal infusion studies have shown LCT results in greater gastric relaxation than MCT [30] and MCT accelerates small-bowel transit time [31], which suggests quicker GE after MCT ingestion; however, our results do not support this. This may be due to the difference in outcome measures or due to the fact the MCT in the current study was eaten as part of a meal and not infused. GE is affected by osmolarity, as the higher the osmolarity of a solution, the greater the delay in GE [32,33]. MCT have a higher osmolarity than LCT, which may drive the difference in GE between MCT and LCT breakfasts in the current study. Furthermore, Beckers et al. [34] administered drinks containing varying amounts of carbohydrate and MCT and found that the drinks with the largest amount of MCT was emptied the fastest; leading to the conclusion that MCT may be emptied faster than LCT, as carbohydrate is emptied faster than LCT. However, the osmolarity of the drinks in that study increased with the increasing carbohydrate content, thus this may be mediated the faster GE after MCT drinks compared to carbohydrate. An in vitro study previously indicated that, due to the water-soluble nature of MCT, MCT are released into the watery environment in the stomach [35], which may delay GE by increasing the osmolarity of the chyme, despite MCT not stimulating the release of CCK. Taken together, the decreased *ad libitum* and 24 h intake may be a combination of two physiological processes during digestion. The first being a delay of GE by the increased osmolarity of MCT, which as discussed, exerts satiating effects. The second then being faster absorption, transport and oxidation than LCT once they have emptied from the stomach; leading to a faster overall absorption despite slower GE.

MCT are known to be unpalatable [36] and may cause gastrointestinal distress [37]. The first study to examine the effect of MCT on satiety reported that fatty acid chain length was linked in a dose-dependent manner with gastric aching [7]. There was no significant effect of oil or food form on nausea in the present study, which may be due to the ingestion of MCT within a mixed meal, as simultaneous MCT with carbohydrate consumption has been shown to abolish adverse effects [38]. Future work could aim to establish the reason for these inconsistent adverse effects, as higher dosages than those administered in the study of Rolls and colleagues [7] have reported no adverse effects [8,9]. 

This study has shown novel findings in that MCT primarily exert a satiating effect when consumed in liquid form; however, there are limitations to consider. In order to provide identical meals (food form notwithstanding), we decided to bake the oils into brownies which were then consumed with ice cream and milk or blended into a milkshake. In one aspect, this is a strength of the study as the meals were identical in both calories and sensory aspects, other than physical form. Furthermore, the LCT needed as part of the brownie was replaced by MCT, thus not adding extra fat. Previous studies have added MCT to meals in order to examine the effect on energy intake compared to LCT [7,8,9,10,11]. Whereas this does allow for direct comparison of LCT and MCT, adding extra energy into the diet is not a logical or feasible method of reducing overall energy intake, thus this is a strength of the current study. However, a limitation of the current study is the use of brownies, as these are not typically consumed breakfast foods, which may alter the satiety response to these foods at this meal [39]. On reflection, given the palatability of the test meal and the positive attributes of meal with regards to the research question, it was deemed suitable for use in the context of this study. Future studies should aim to use more contextually appropriate foods in order to produce more ecologically valid results. 

## 5. Conclusions

Taken together, these results demonstrate that MCT lead to reduced subsequent energy intake, resulting in decreased overall 24 h intake, compared to an isocaloric meal containing LCT. We have shown, for the first time, that this may be dependent on the form in which MCT are ingested, as MCT ingested in solid form do not appear to elicit a stronger satiating effect. We propose this is due to the delayed GE of MCT, as MCT delivered in liquid form was delayed to the same extent as MCT delivered in solid form. This does explain why MCT leads to decreased intake compared to LCT in liquid form, however it is not clear why there was no difference in GE between MCT meals of solid or liquid form. Further research should aim to elucidate these mechanisms, as well as provide more information on the conditions in which MCT cause adverse effects. 

## Figures and Tables

**Table 1:** Gastric emptying (GE) half time, lag phase, latency phase and ascension time of all participants (*n* = 29). Values are means ± SD.

GE Parameter	Con-S	Con-L	MCT-S	MCT-L
Half time (min)	134.3 ± 33.8	122.4 ± 34.1	157.8 ± 32.7 ^1,2^	173.0 ± 49.8 ^1,2^
Lag phase (min)	55.0 ± 13.9	46.6 ± 11.0 ^3^	61.8 ± 10.2	60.7 ± 12.3
Latency phase (min)	50.0 ± 19.9	37.4 ± 11.5 ^3^	52.9 ± 17.5	43.8 ± 10.1
Ascension time (min)	168.4 ± 38.5	165.7 ± 64.4	189.9 ± 38.1 ^2^	216.0 ± 51.0 ^1,2^

Abbreviations: Con-S, control oil, solid form; Con-L, control oil, liquid form; MCT-S, MCT oil, solid form; MCT-L, MCT oil, liquid form. ^1^ Significantly different from Con-S, ^2^ significantly different from Con-L, ^3^ significantly different from all other trials. Significance accepted at the *p* < 0.05 level.

## Supplementary Materials

The following are available online at https://www.mdpi.com/2072-6643/11/7/1638/s1, Supplementary Material S1: Diagram indicating flow of participants through the study.
