# Supplementary figures and images for: Food Intake and Satiety Response after Medium-Chain Triglycerides Ingested as Solid or Liquid
Source: Nutrients. 2019 Jul 17;11(7):1638. doi: 10.3390/nu11071638 (PMC6683029; doi:10.3390/nu11071638)

Supplementary material 1: Diagram indicating flow of participants through the study.

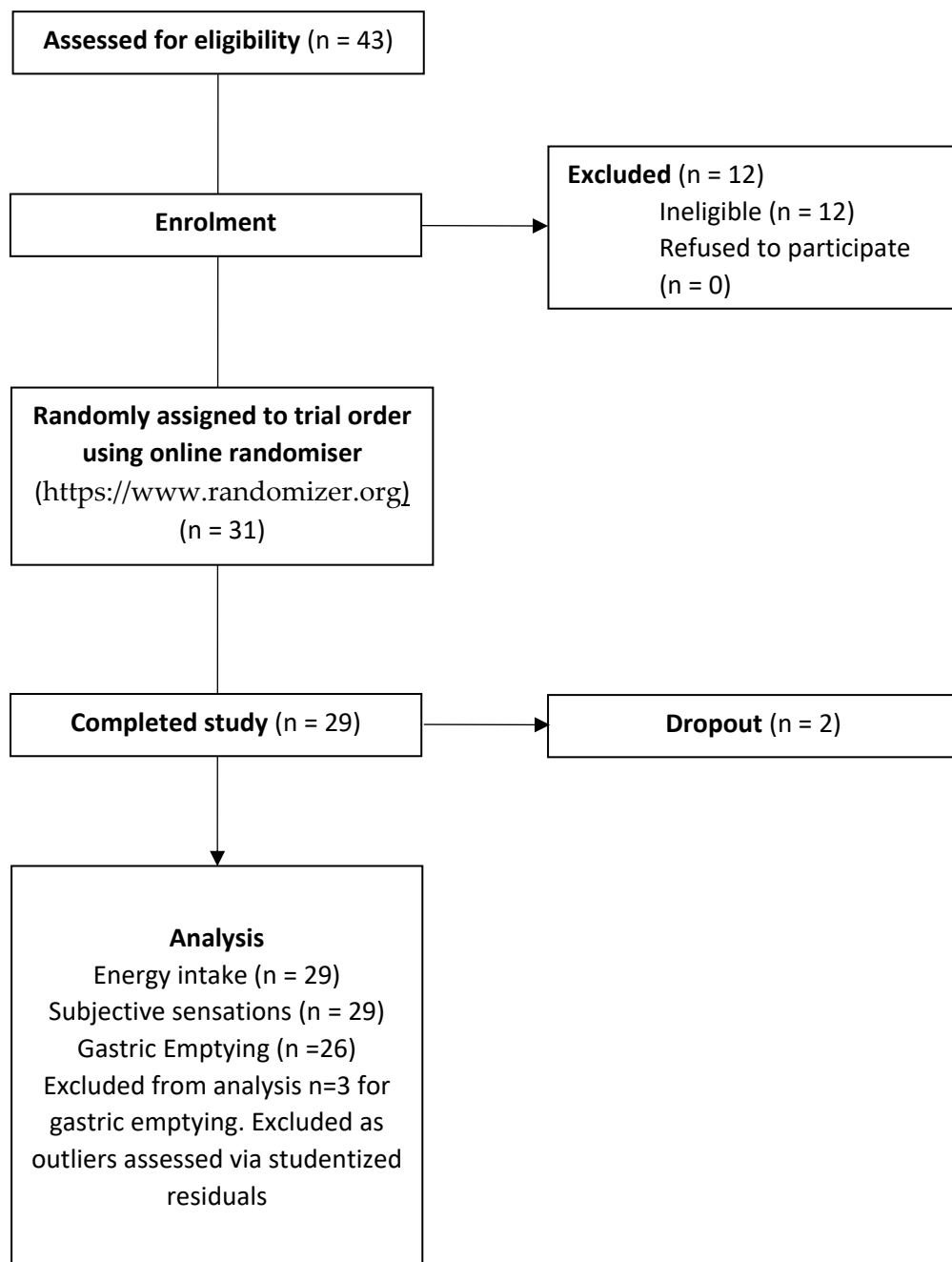

Supplement: Supplementary file 1 [file nutrients-11-01638-s001.pdf]
